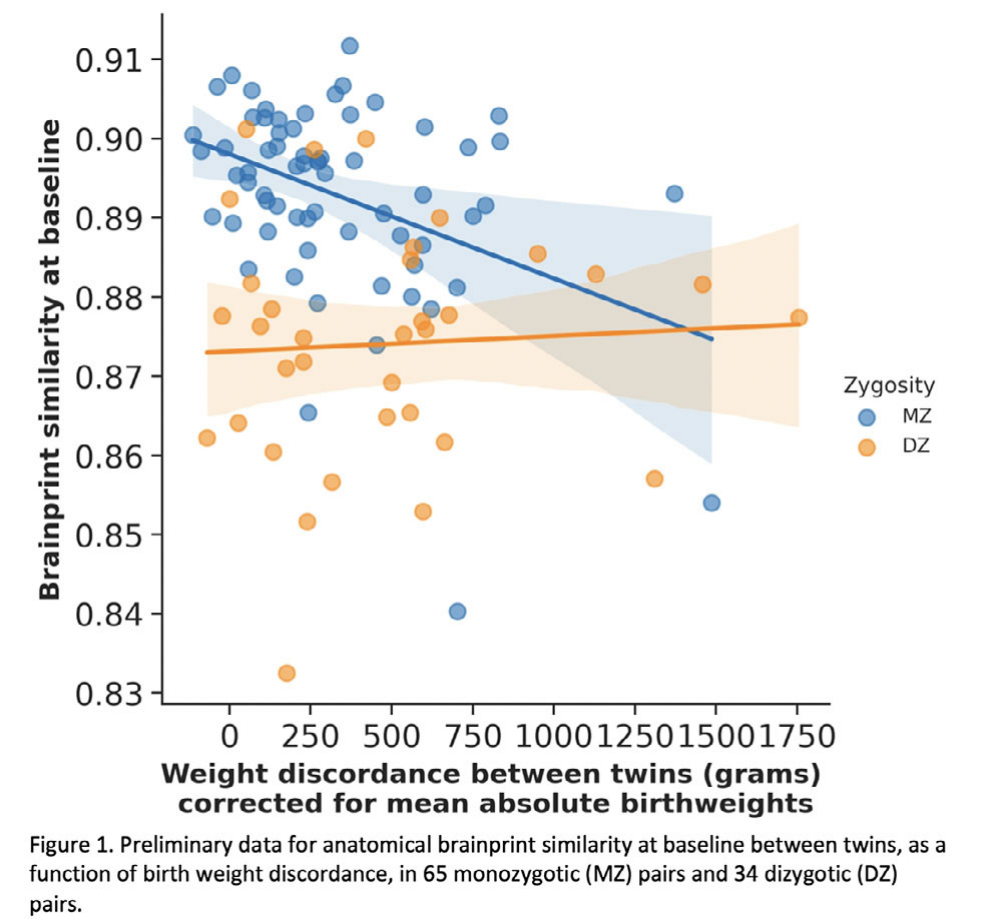# An experimental approach to disentangle neurodevelopmental and current factors of influence on brain and cognition: an intervention twin study

**DOI:** 10.1002/alz.088714

**Published:** 2025-01-09

**Authors:** Kristine B Walhovd, Sneve H Markus, Øystein Sørensen, Anne Cecilie Sjøli Bråthen, Emilie Sogn Falch, Pablo F. Garrido, Jonas Kransberg, Maksim Slivka, José‐Luis Alatorre‐Warren, Knut Overbye, Nikolai Olavi Czajkowski, Anders M Fjell

**Affiliations:** ^1^ LCBC, University of Oslo, Oslo Norway; ^2^ University of Oslo, Oslo Norway; ^3^ University of Oslo, Oslo, Oslo Norway

## Abstract

**Background:**

Neurodevelopmental origins of functional variation through the lifespan are acknowledged, but pathways need to be identified. The objectives of the project Set‐to‐change is to test whether and how early life environmental factors and genetic makeup regulate brain and cognition and its change, as well as neurocognitive plasticity in response to training through the lifespan.

**Method:**

Preliminary analyses for the first months are presented. We investigate differences in brain and cognition and their change, in adult mono‐ (MZ) and dizygotic (DZ) twins (total n ∼220 individuals, age range 16‐79 years, mean 36), with varying degrees of prenatal environmental variance, as indexed by their extent of discordance in birth weight (BW). Half of the sample trained in a novel navigation intervention utilizing true locomotion in virtual reality, assessed with brain MRI and cognitive measures at pre‐ and post‐ 10 weeks intervention in an AB/BA crossover design. Effects of training on hippocampus volume are analyzed using generalized additive mixed models (GAMM4) accounting for condition (pre‐train, post‐train, post‐rest), sex, age (smooth), time, and time x age. Anatomical brainprints from T1 and T2W scans are used to assess effects of genetic (MZ/DZ) and early environmental (birth weight; BW) differences.

**Results:**

Preliminary analyses indicate a positive effect of training on hippocampal volumes. Differences in behavioral level reached in training, related to differences in BW among MZ twins, but the higher BW twin tended to train more. Using “brainprints” (Valizadeh et al. Sci Rep 2018, 8, 5611) from multicontrast MRI data, we observed that across 10 weeks, self‐similarity was high and could be used for perfect identification of one self and MZ twin, DZ‐similarity also identifiable. Brainprint similarity at baseline varied as a function of BW in MZ twins (Figure 1). BW discordance in MZ twins (n for analysis: 27 pairs) related to degree of functional plasticity, as measured by more whole brain connectome change after training being higher with higher BW.

**Conclusion:**

Twin designs sampling early and current life environmental conditions experimentally, may further enhance our understanding of the impact of early life factors on current outcomes and in adulthood.